# Practical considerations for the use of IL-23p19 inhibitors in inflammatory bowel disease: how to choose between them and why it matters?

**DOI:** 10.1093/ecco-jcc/jjaf144

**Published:** 2025-08-01

**Authors:** Cecilia Lina Pugliano, Raymond Fueng-Hin Liang, Andrea Ruffa, Marietta Iacucci, Subrata Ghosh

**Affiliations:** APC Microbiome Ireland, College of Medicine and Health, University College Cork, Cork, Ireland; APC Microbiome Ireland, College of Medicine and Health, University College Cork, Cork, Ireland; APC Microbiome Ireland, College of Medicine and Health, University College Cork, Cork, Ireland; APC Microbiome Ireland, College of Medicine and Health, University College Cork, Cork, Ireland; APC Microbiome Ireland, College of Medicine and Health, University College Cork, Cork, Ireland

**Keywords:** Guselkumab, inflammatory bowel diseases, mirikizumab, risankizumab, anti-IL23p19

## Abstract

A wide range of advanced therapies has become available in recent years for the treatment of moderate-to-severe inflammatory bowel disease (IBD). Among these, monoclonal antibodies targeting the interleukin 23 p19 subunit (anti-IL23p19) have emerged as a promising therapeutic class. Pivotal Phase 3 trials have demonstrated their favorable clinical efficacy and safety in both Crohn’s disease (CD) and ulcerative colitis (UC). Three such agents, Risankizumab, Mirikizumab, and Guselkumab, have now been approved in CD and UC. For gastroenterologists, the ability to rationally select among these options to personalize treatment and maximize patient benefit is critical. Key factors to consider when selecting an anti-IL23p19 agent include patient preference regarding mode of administration, IBD phenotype, presence of coexisting extra-intestinal manifestations, concomitant immune-mediated diseases, and previous advanced-therapy exposure. Our review summarizes the current clinical evidence on anti-IL23p19 therapies and provides practical guidance on their use in IBD clinical management, including dosing strategies, choice of dose in CD and UC, and clinical positioning across patients. Finally, anti-IL23p19 inhibition may represent a future first-line therapy option for moderate-to-severe IBD, particularly in patients with concomitant IL-23 driven comorbidities such as psoriasis. Its use in combination with other advanced therapies in selected patients is being explored to enhance therapeutic efficacy and improve long-term outcomes. Further real-world studies are needed to assess its effectiveness and benefits in complex disease phenotypes, including perianal fistulizing Crohn’s disease.

## 1. Introduction

Despite the complex and still not fully understood pathogenesis of inflammatory bowel disease (IBD), immune dysregulation is undoubtedly a key driver of disease onset and progression. The advent of advanced therapies, selectively targeting immune pathways involved in the initiation, persistence, and progression of inflammation, has revolutionized management of both ulcerative colitis (UC) and Crohn’s disease (CD).^1–3^

**Figure 1.**
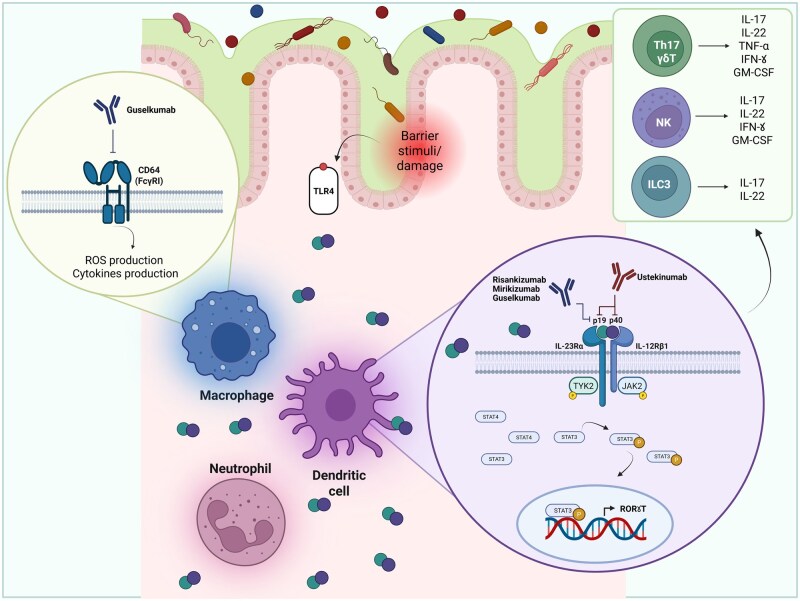
Schematic representation of IL-23 structure and signaling pathway in intestinal mucosa. IL-23 is a two-part cytokine made up of p19 and p40 subunits, primarily produced by immune cells like macrophages and dendritic cells in response to microbial signals. It works by binding to a specific receptor complex that activates JAK2 and TYK2 kinases, which then activate STAT3. This leads to the nuclear translocation of STAT3 and the expression of RORγt, a key transcription factor involved in inflammatory responses. The main function of IL-23 is to maintain and expand Th17 cells; however, it also acts on various other immune cells, including NK T cells, ILC3 cells, and macrophages. Through these interactions, IL-23 promotes the production of multiple inflammatory molecules like IL-17, IL-22, TNF-α, and antimicrobial peptides. Overall, IL-23 serves as a central driver of sustained pro-inflammatory immune responses in the body. Anti-IL23p19 agents selectively bind the subunit p19 of IL23 cytokine, while anti-IL12/23 agents work on both subunits p19 and p40. Additionally, Guselkumab can bind to CD64 receptors on myeloid cells, including macrophages, monocytes, and dendritic cells. This interaction weakens downstream inflammatory signaling, providing an additional mechanism beyond direct IL-23 neutralization for controlling inflammation. Created in “BioRender.com.” GM-CSF: granulocyte-macrophage colony-stimulating factor; NK: natural killer; IFN-γ: interferon γ; ILC3: type 3 innate lymphoid cells; JAK2: Janus kinase 2; RORγt: retinoic acid receptor-related orphan receptor gamma transcription factor; STAT3: signal transducer and activator of transcription 3; TLR4: toll-like receptor 4; TNF-α: tumor necrosis factor α; TYK2: tyrosine kinase 2.

Agents directed against interleukin 23 (IL-23) signaling have gained particular prominence in recent years. IL-23 is a heterodimeric cytokine and comprises p19 and p40 subunits, which together bind a receptor complex formed by IL-23Rα. IL-23 signaling activates JAK2/TYK2 kinases and downstream STAT3/4 transcription factors, inducing the expression of pro-inflammatory mediators such as IL-17A, IL-22, and IFN-γ.^2^^,^^4^  Figure 1 illustrates the IL-23 signaling pathway in detail.

The first successful biologic to exploit this pathway was Ustekinumab (UST), a monoclonal antibody targeting the p40 subunit shared by IL-12 and IL-23. ^5^^,^^6^ Subsequent research, however, has emphasized the predominant pathogenic role of IL-23 rather than IL-12.^1^ This led to the development of more selective IL-23 inhibitors, a next-generation of monoclonal antibodies targeting only the p19 subunit, including Risankizumab (RIS; IgG1), Mirikizumab (MIR; IgG4), and Guselkumab (GUS; IgG1-λ). Inhibition of IL-12, on the other hand, may confer minor susceptibility to some pathogens.

Notably, GUS offers an additional unique property: it binds to CD64 (FcγRI), a receptor highly expressed on myeloid cells (Figure 1). CD64+ myeloid cells are found in elevated numbers within the inflamed colonic tissue of patients with IBD. By engaging these cells through its Fc region, while simultaneously selectively neutralizing IL-23, GUS may offer enhanced therapeutic potential. ^7–9^ However, the current clinical relevance of this dual mechanism remains incompletely understood. ^7^^,^^8^ The availability of multiple IL-23p19 inhibitors, all with comparable efficacy and safety profiles, has introduced a new challenge in daily clinical practice: choosing the most appropriate agent for each patient. This review aims to provide a concise overview of the current landscape of selective IL-23p19 inhibitors in IBD, highlighting both shared and unique features and offering a practical, patient-centred guidance for clinical decision-making.

## 2. Methods

A comprehensive literature search was conducted using the PubMed MeSH database for English-language publications up to May 2025. Validated search strategies and keywords included terms such as “inflammatory bowel disease,” “Crohn’s disease,” “ulcerative colitis,” “interleukin-23,” “Guselkumab,” “Risankizumab,” “Mirikizumab,” “Ustekinumab,” “dual biologic,” and “advanced combination therapy.” Studies were selected based on relevance and study design, with priority given to clinical trials and meta-analyses, followed by prospective and retrospective observational studies. Pediatric studies and non-English publications were excluded. Additionally, reference lists of selected articles were manually screened to ensure that no relevant studies were missed during the electronic search.

## 3. Clinical applications of anti-IL23 therapies

**Figure 2.**
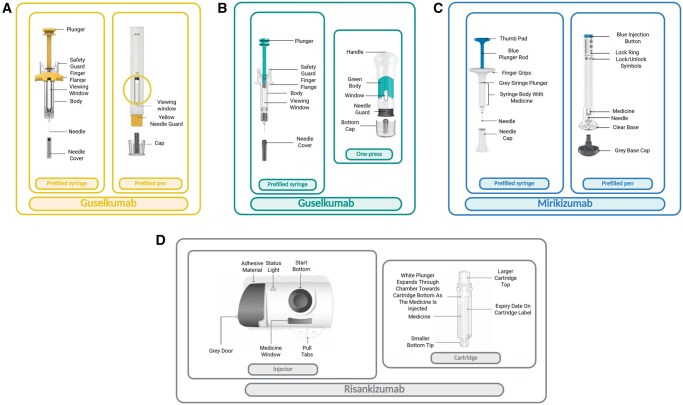
Representative subcutaneous devices for anti-IL-23p19 therapies. The figure illustrates different administration devices for approved anti-IL23 monoclonal antibodies: Guselkumab (GUS), Mirikizumab (MIR), and Risankizumab (RIS). Key device characteristics are summarized, including device type (prefilled syringe, prefilled pen, one press injection, or autoinjector with its cartridge) and design features intended to improve adherence for patients. Information is based on regulatory-approved product labelling and publicly available data. **(A)** Prefilled syringe and prefilled pen for GUS (200 mg); **(B)** prefilled syringe and one-press injection for Guselkumab (100 mg); **(C)** prefilled syringe and prefilled pen for MIR (100 mg or 200 mg); **(D)** on-body injector and drug cartridge for RIS (180 mg or 360 mg). Created in “BioRender.com.”

Three IL-23p19 subunit inhibitors (anti-IL23p19) agents, GUS, RIS, and MIR, have been recently approved by the European Medicines Agency and United States Food and Drug Administration for the treatment of moderate-to-severe CD and UC. Their efficacy has been proven through conventional intravenous (IV) induction (three doses administered 4 weeks apart in an infusion center) followed by subcutaneous (SC) maintenance dosing administered every four to eight weeks.^10–17^  Figure 2 illustrates a comparison of the available subcutaneous devices for anti-IL23p19 agents.

### 3.1 GUS and its unique SC induction in IBD management

**Figure 3.**
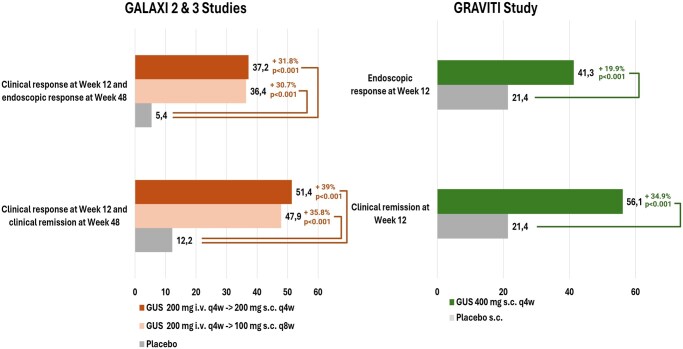
Comparative graph for intravenous and subcutaneous Guselkumab induction dosing. The figure compares the two major intravenous (GALAXI 2 & 3) and subcutaneous (GRAVITI) induction study protocols for Guselkumab. Specifically, it highlights the non-inferiority of subcutaneous induction compared with intravenous in terms of clinical remission (w12) and endoscopic response rates (w48) among the overall study population. Created in ”BioRender.com.”

Two recent phase 3 double-blind placebo (PBO)-controlled trials demonstrated that SC induction with GS is not inferior to IV induction, showing comparable clinical remission and endoscopic response rates at week 12 (Figure 3). These findings established GUS as the first anti-IL23p19 agent with proven efficacy for both SC induction and maintenance therapy in CD and UC.

**Figure 4.**
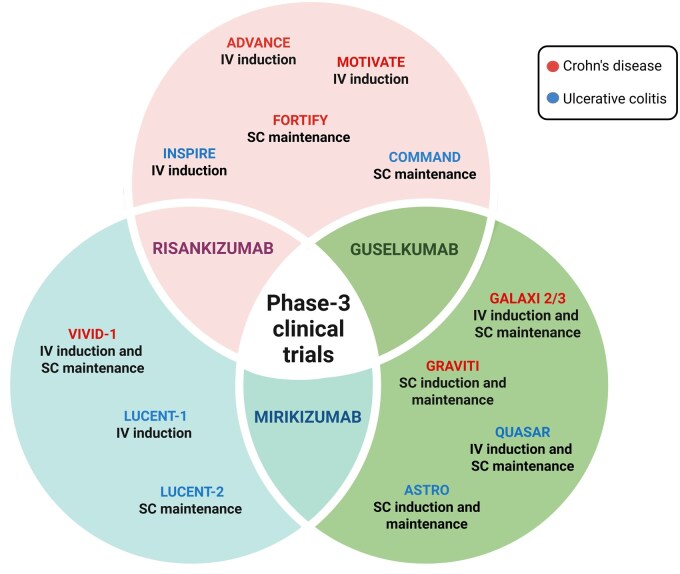
Pivotal regulatory trials for IL-23p19 inhibitors. The figure presents the main Phase 3 trials for the anti-IL-23p19 agents, respectively, in green (Guselkumab), blue (Mirikizumab), and pink (Risankizumab). Crohn’s disease trials are bold in red, while blue text indicates Ulcerative Colitis trials. Created in ”BioRender.com.”

- **GRAVITI trial (** Figure 4 **)—moderate-to-severe CD** ^18^
** Dosing regimen:** 400 mg SC in single dose, chosen to approximate the established 200 mg IV dose used in the previous GALAXI 2/3 trials^17^ (estimated 50% bioavailability for the SC formulation)
** Endpoints vs PBO at week 12:** both SC and IV formulations achieved similar improvements over PBO in terms of clinical remission (SC 56.1% vs 21.4% [*P* <.001]; IV 47.1% vs 18.9% [*P* <.001]) and endoscopic response (SC 41.3% vs 21.4% [*P* <.001]; IV 36.9% vs 12.2% [*P* <.001]).
- **ASTRO trial (** Figure 4 **)—moderate-to-severe UC** ^19^
** Dosing regimen:** 400 mg SC every 4 weeks in three doses (w 0,4,8)
** Endpoint vs PBO at week 12:** A significantly higher proportion of patients achieved clinical remission over PBO. Mirrored data when compared with the IV QUASAR study^15^ (SC 27.6% vs 6.5% [*P* <.001]; IV 23% vs 8% [*P* <.0001]).

The availability of a SC induction regimen represents a significant alternative choice in the therapeutic armamentarium for IBD management. It offers the potential to improve patient adherence, enhance access to treatment, reduce the burden and cost on infusion services, and minimize the risks associated with IV-related infusion reactions.

### 3.2 Personalizing anti-IL23 therapeutic regimen

**Figure 5.**
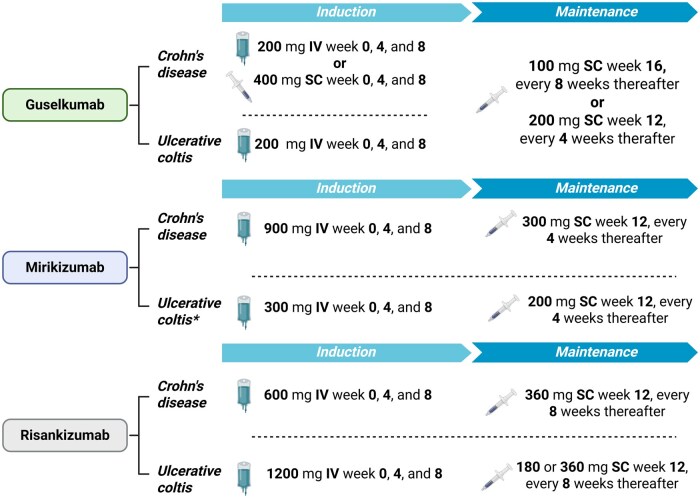
Schematic overview of induction and maintenance doses for anti-IL-23 therapies. The figure illustrates induction and maintenance dosing regimens of approved anti-IL23 monoclonal antibodies. For Guselkumab, induction can be achieved with either 200 mg intravenously (IV) in ulcerative colitis (UC) and Crohn’s disease (CD) or 400 mg (two 200 mg pre-filled syringes or two 200 mg pre-filled pens) subcutaneously (SC) only in CD. The recommended maintenance dose is 100 mg (via one 100 mg pre-filled syringe or press injection) SC every 8 weeks. In patients with suboptimal response after induction, a higher maintenance dose of 200 mg (one 200 mg pre-filled syringe or pen) SC every 4 weeks may be considered. For Mirikizumab, the maintenance dose is 200 mg SC (two 100 mg pre-filled syringes or pen) every 4 weeks for UC, while 300 mg SC (one 100 mg pre-filled syringe or pen and one 200 mg pre-filled syringe or pen) every 4 weeks is recommended for CD. * UC patients with inadequate response after 12 weeks of induction may receive extended induction dosing (300 mg IV at weeks 12, 16, and 20). Responders can transition to subcutaneous maintenance dosing (200 mg every 4 weeks) from week 24. In the event of a loss of response during maintenance, an IV re-induction (300 mg every 4 weeks for up to 3 doses) may be attempted, followed by the resumption of subcutaneous therapy if clinical benefit is regained. The maintenance dose of Risankizumab in UC is 180 mg SC every 8 weeks, which can be increased to 360 mg SC every 8 weeks for patients with inadequate response after induction period. Created in ”BioRender.com.”

Each approved anti-IL23 agent has a defined induction and maintenance regimen for treating moderate‑to‑severe UC or CD (Figure 5). Typically, therapy begins with an induction phase (often involving three IV doses administered at Weeks 0, 4, and 8), followed by a maintenance phase with regular SC injections every 4–8 weeks. The exact schedule depends on the agent and clinical indication.

Notably, RIS and MIR vary not only in their dosing schedules between UC and CD, but also between the induction and maintenance phases (Figure 5). In contrast, GUS offers a maintenance regimen that is identical for UC, CD, and plaque psoriasis/psoriatic arthritis. Specifically, the 100 mg SC every-8-weeks regimen aligns with the psoriasis dosing schedule protocol (100 mg SC at Weeks 0 and 4, followed by every-8-week maintenance), providing a significant advantage in terms of treatment simplicity and suitability for patients with coexistent conditions.

## 4. Anti-IL23p19 vs anti-IL12/23 in moderate-to-severe CD

### 4.1 GUS

Overall, SC GUS maintenance regimen significantly improved clinical remission and endoscopic responses rates compared with UST, even in patients with prior inadequate biologic response (bio-IR).

- **GALAXI 2/3 (** Figure 4 **) trials** ^20^

**Dosing regimen:** SC GUS 100mg every 8 weeks (q8w) or 200mg every 4 weeks (q4w) vs SC UST

**Major secondary endpoints vs UST**: superiority of SC GUS across major endpoints, including the composite endpoint of clinical remission and endoscopic response (SC GUS 100mg q8w 37.3% [*P* =.032] and 200 mg q4w 40.8% [*P* =.005] vs SC UST 25.6%). Superiority was maintained among the bio-IR subgroup. Recent data has affirmed the superior efficacy and comparable safety of GUS to UST up to Week 48.

### 4.2 RIS

RISA was compared head-to-head with UST in CD, but not in UC.

- SEQUENCE trial^21^
  Open-label head-to-head trial comparing RIS vs UST in Anti-TNF-experienced CD patients with moderate-to-severe disease.
** Endpoints:** non-inferiority to UST in terms of clinical remission (58.6% vs 39.5%, adjusted difference 18.4%), and superiority to UST in terms of endoscopic remission (31.8% vs 16.2%, adjusted difference 15.6%).
- **ADVANCE, MOTIVATE (induction)** ^14^  **and FORTIFY (maintenance)**^13^  **trials (**Figure 4**)**
  These trials did not have UST comparator arms but included bio-IR and patients with previous UST failure.
** Endpoints vs PBO:** Both induction doses in ADVANCE/MOTIVATE significantly increased clinical remission and endoscopic response in the overall and bio-IR subpopulations. In FORTIFY, both maintenance doses outperformed PBO similarly in terms of clinical remission and endoscopic response.

### 4.3 MIR

MIR demonstrated non-inferiority to UST for both induction and remission phases, without significant differences in bio-IR subgroups. However, superiority in endoscopic response was not achieved.

- **VIVID-1 trial (** Figure 4 **)** ^11^

Head-to-head trial comparing MIR vs UST.

**Endpoints:** MIR non-inferior to UST for clinical remission (54.1% vs 48.4% [*P* <.0001]) and did not achieve endoscopic response superiority. In the bio-IR population, numerically—but not statistically significant—higher proportions of clinical remission and endoscopic response among MIR-treated individuals compared to UST.

## 5. Anti-IL23p19 vs anti-IL-12/23 in moderate-to-severe UC

No comparative trials between anti-IL23p19 and anti-IL12/23 agents are currently available in UC. Notwithstanding trial differences, indirect comparisons suggest overall higher clinical remission rates in GUS compared with UST.

- **QUASAR (IV GUS 200mg) vs UNIFI (IV UST 130mg)** ^15^ ^,^ ^22^
** Endpoints vs PBO at week 8:** higher clinical remission rates in QUASAR (40% vs 21% [*P* <.0001)]) compared with UNIFI (15.6% vs 5.3% [*P* <.001])
- **QUASAR (SC GUS 100mg q8w or 200mg q4w) vs UNIFI (SC UST 90mg q12w or 90mg q8w)** ^15^ ^,^ ^22^

**Endpoints vs PBO at week 44**: GUS showed better response in QUASAR (100mg q8w 45% vs 200mg q4w 50% vs PBO 19% [*P* <.0001]) compared to UST maintenance in UNIFI (90mg q12w 38.4% [*P* =.002], vs 90mg q8w 43.8% [*P* <.001], vs PBO 24%).

## 6. Evaluating the safety of anti-IL23p19 inhibition

No major safety concerns have emerged from the major anti-IL23p19 trials for IBD (detailed in Figure 4).

Two systematic reviews and meta-analyses have demonstrated a favorable safety profile comparable to PBO, in both CD (serious adverse events in induction, risk ratio [RR] 0.55 [0.44, 0.73] and maintenance RR 0.72 [0.53-0.98]) and UC (adverse events in induction RR 0.94 [0.86,1.02] and maintenance RR 0.93 [0.86-0.99]).^23^ Published Phase 3 trials have corroborated these findings.^11^^,^^12^^,^^15^^,^^20^^,^^24^

## 7. Choosing between anti-IL23p19 agents—specific clinical considerations and therapeutic positioning

### 7.1 Advanced therapy-experienced patients

All these Phase 3 PBO-controlled trials of anti-IL23p19 agents in IBD included patients with prior exposure to advanced therapy.^11^^,^^12^^,^^15^^,^^20^^,^^24^ Among them, the therapeutic efficacy of anti-IL23p19 agents demonstrated superiority over PBO.

For CD, participants had previously received mostly anti-TNF agents and/or Vedolizumab, with the rare exception of UST failure. All three agents achieved significantly higher clinical remission rates at week 12 compared to PBO, with GUS showing the most pronounced treatment effect (IV 46.0% vs 19.2% PBO; SC 60.2% vs 17.0% PBO). For maintenance of endoscopic remission, anti-IL23p19 agents maintained their therapeutic advantage over PBO, with the higher-dose subcutaneous GUS regimen (200mg q4w) demonstrating the best efficacy at week 48 (37.7% vs 0% PBO; 24.3, 50.5).^18^

For UC, enrolled patients had previously received biologic agents or JAK inhibitors, with only a few studies including sphingosine 1-phosphate inhibitor exposure. Anti-IL23p19 agents similarly outperformed PBO for week 12 clinical remission, with SC GUS showing the largest treatment differential (16.1% vs 3.6% PBO). During maintenance phases, all agents achieved superior remission rates compared to placebo, with IV GUS demonstrating significant clinical and endoscopic remission at week 44 (*P* <.0001), while RIS and MIR showed enhanced efficacy at week 52 (*P* <.001 for RIS 180 mg and *P* <.002 for RIS 360 mg) and 40 (*P* <.001) respectively.^10^^,^^12^^,^^15^

Tables 1 and 2 summarize the differential trial outcomes between bio-naïve and bio-IR patients in CD and UC, respectively.

**Table 1.** Comparative CD trial outcomes (drug vs PBO) by biologic/advanced therapy exposure status.

	W12 clinical remission		W12 endoscopic response		W48 clinical remission		W48 endoscopic response		W48 endoscopic remission		W52 clinical remission		W52 endoscopic remission	
	Bio-naïve	Bio-IR	Bio-naïve	Bio-IR	Bio-naïve	Bio-IR	Bio-naïve	Bio-IR	Bio-naïve	Bio-IR	Bio-naïve	Bio-IR	Bio-naïve	Bio-IR
**GUS IV induction** **(GALAXI 2, 3)**	49.6% vs 16.4%, *P *<.001	46.0% vs 19.2%, *P *<.001	46.3% vs 18.0%, *P *<.001	29.0% vs 6.4%, *P *<.001	200mg 54.7% vs 100mg 51.7% vs PBO 11.5%, *P *<.001*	200mg 49.7% vs 100mg 45.8% vs PBO 12.8%, *P *<.001*	200mg 43.8% vs 100mg 40.5% vs PBO 6.6%, *P *<.001*	200mg 31.3% vs 100mg 35.9% vs PBO 5.1%, *P *<.001*						
**GUS SC induction** **(GRAVITI)**	49.5% vs 25.0%, *P *<.05	60.2% vs 17.0%, *P *<.05	48.6% vs 26.8%, *P *<.05	33.3% vs 17.0%, *P *<.05	100mg q8w 62.3% vs 200mg q4w 67.3% vs PBO 23.2%, *P *<.05	00mg q8w 58.2% vs 200mg q4w 62.3% vs PBO 9.4%, *P *<.05*			100mg q8w 41.5% vs 200mg q4w 42.3% vs PBO 10.7%, *P *<.05	100mg q8w 20.0% vs 200mg q4w 37.7% vs PBO 0.0%, *P *<.05				
**RIS**	ADVANCE: 600mg 48.9% vs 1200mg 47.1% vs PBO 23.1%**	ADVANCE: 600mg 42.6% vs 1200mg 37.7% vs PBO 25.8%**	ADVANCE: 600mg 50.4% vs 1200mg 43.6% vs PBO 12.8%**	ADVANCE: 600mg 32.8% vs 1200mg 23.6% vs PBO 11.3%**							FORTIFY: 180mg 72.7% vs 360mg 64.1% vs PBO 58.5%**	FORTIFY: 180mg 48.7% vs 360mg 48.0% vs PBO 35.0%**	FORTIFY: 180mg 52.3% vs 360mg 48.7% vs PBO 22.0%**	FORTIFY: 180mg 21.2% vs 360mg 35.4% vs PBO 9.8%**
**MIR** **(VIVID-1)**	39.6% vs 25.5%, *P *<.01	35.6% vs 24.7%**	37.9% vs 16.7%, *P *<.0001	26.7% vs 8.2%, *P *<.0001							56.7% vs 26.5%, *P *<.0001	51.2% vs 12.4%, *P *<.0001	18.5% vs 2.9%, *P *<.0001***	13.2% vs 1.0%, *P *<.0001***

* Co-primary endpoint with W12 clinical response.

** No significance testing performed.

***I n W12 clinical responders.

**Table 2.** Comparative UC trial outcomes (drug vs PBO) by biologic/advanced therapy exposure status.

	W12 clinical remission		W12 endoscopic improvement		W44 clinical remission		W44 endoscopic remission		W52 clinical remission		W52 endoscopic remission	
	Bio-naïve	Bio-IR	Bio-naïve	Bio-IR	Bio-naïve	Bio-IR	Bio-naïve	Bio-IR	Bio-naïve	Bio-IR	Bio-naïve	Bio-IR
**GUS IV induction** **(QUASAR)**	32% vs 12%, *P *<.0001	12% vs 4%, *P *=.0052	38% vs 17%, *P *<.0001	15% vs 5%, *P *=.0048	100mg q8w 50% (*P *=.0002) vs 200mg q4w 58% (*P *<.0001) vs PBO 26%	100mg q8w 40% vs 200mg q4w 40% vs PBO 8% (*P *<.0001)	100mg q8w 38% (*P *=.0053) vs 200mg q4w 42% (*P *=.0044) vs PBO 20%	100mg q8w 31% (*P *=.0006) vs 200mg q4w 24% (*P *=.0046) vs PBO 8%				
**GUS SC induction** **(ASTRO)**	36.0% vs 8.9%, *P *<.001	16.1% vs 3.6%, *P *<.01	45.7% vs 17.7%, *P *<.001	24.1% vs 7.1%, *P *<.01								
**RIS** **(INSPIRE, COMMAND)**	29.7% vs 8.4%, Δ 21.3% [14.6%, 27.9%]	11.4% vs 4.3%, Δ 7.2% [2.6%, 11.8%]	47.6% vs 14.2%, Δ33.4% [25.7%, 41.2%]	25.9% vs 10.1%, Δ15.8% [9.2%, 22.3%]					180mg 50.9% (Δ19.8% [-0.2%, 39.7%]) vs 360mg 61.7% (Δ30.6% [11.2%, 50.0%] vs PBO 31.1%	180mg 36.6% (Δ13.4 [2.6%, 24.2%]) vs 360mg 29.5% (Δ6.3% [-4.0%, 16.7%]) vs PBO 23.2%	180mg 36.6% (Δ16.6% [-1.8%, 35.0%]) vs 360mg 51.6% (Δ31.6% [13.1%, 50.2%]) vs PBO 20.0%	180mg 18.7% (Δ5.6% [-3.1%, 14.3%]) vs 360mg 15.1% (Δ2.1% [-6.1%, 10.3%]) vs PBO 13.0%
**MIR** **(LUCENT 1, 2)**	30.9% vs 15.8%	15.2% vs 8.5%							51.5% vs 30.7%	46.1% vs 15.6%	62.4% vs 34.2%	50.8% vs 20.3%

Δ: between-group difference.

### 7.2 Non-IBD immune-mediated diseases

In moderate-to-severe psoriasis, RIS showed better outcome including more rapid response and greater effect persistence compared with UST. Moreover, GUS demonstrated effectiveness in treatment of UST-resistant psoriasis. Both RIS and GUS have established efficacy in psoriatic arthropathy.^25–28^

### 7.3 Perianal disease

Currently, no published data are available on anti-IL23p19 agents for perianal fistulizing CD. The ongoing phase 3 FUZION CD trial (NCT05347095) is investigating the efficacy of GUS in this population through a randomized, placebo-controlled design. Results from this study are anticipated to provide pivotal evidence to inform the management of this complex disease phenotype.^29^

### 7.4 Proctitis

Despite its limited anatomical extent, ulcerative proctitis (UP) can present severe, treatment-refractory symptoms. Major clinical trials evaluating advanced therapies for UC have historically excluded patients with "proctitis-only" disease, resulting in a significant evidence gap. Specifically, no trials of anti-IL23p19 agents have focused on or stratified outcomes for patients with UP. Thus, the current use of this therapeutic class in UP remains empirical. Nonetheless, emerging data are encouraging. A recent case report described clinical success using a combination of MIR and Upadacitinib in a patient with refractory UP, highlighting the potential role of anti-IL23p19 agents in selected, challenging UP clinical scenarios.^30^

### 7.5 Targeting anti-IL23p19 early as first-line in IBD

Anti-IL-23p19 agents have emerged as a promising therapeutic class, demonstrating favorable safety and immunogenicity profiles that position them as potential first-line treatments for moderate-to-severe IBD, with added clinical value for patients presenting concurrent IL-23-driven inflammatory comorbidities such as psoriasis or psoriatic arthritis. However, critical evidence gaps remain regarding their efficacy in challenging clinical scenarios, including perianal fistulizing disease for first line use.

## 8. Future directions—combination therapies and the rise of personalized medicine in IBD

Despite recent advancements, current IBD therapies still face significant limitations. Approximately, around 50% of patients fail to achieve clinical remission with monotherapy, and among those who initially respond, durability is limited. Repeated treatment switches further reduce the chances of achieving a sustained remission. Additionally, therapies effective for intestinal symptoms may be less effective for managing extra-intestinal manifestations of the disease.^31–33^

Advanced combination therapy (ACT), which leverages distinct mechanisms of action, offers a strategy to overcome treatment resistance and surpass the current therapeutic ceiling that limits treatment effectiveness in IBD.^31^ The newer selective anti-IL23 agents may represent a well-positioned pharmacological class for combination approaches due to their favorable safety and efficacy profiles, making them ideal candidates for multi-targeting therapeutic strategies.

A landmark randomized controlled trial (VEGA) by Feagan and colleagues provided interesting evidence for dual biological therapy in moderate-to-severe UC. Among 214 biologic-naïve patients, 71 treated with combination induction therapy (GUS plus Golimumab) demonstrated a significantly high rates of clinical remission and endoscopic response compared to those receiving either agent alone. Notably, this enhanced efficacy was achieved without compromising safety.^34^ These findings represented a breakthrough in IBD management, providing the first robust evidence that dual biological therapy may be both feasible and superior to monotherapy for achieving comprehensive disease control.

Looking ahead, ongoing studies such as DUET-CD and DUET-UC are evaluating dual biologic therapy, which holds the potential to open the doors to the era of anti-IL-23-driven ACT in IBD. These phase 2b randomized, double-blind, placebo-controlled trials, respectively involving patients with moderate-severe CD and UC, are evaluating clinical remission and mucosal healing outcomes comparing GUS plus Golimumab combination therapy at different dosages (high, mild and low) versus monotherapy of each agent alone and placebo (ClinicalTrials.gov identifiers: NCT05242471 DUET-CD and NCT05242484 DUET-UC).^35^^,^^36^

The DUET trials represent a critical step toward establishing ACT as a standard treatment approach to test the therapeutic ceiling of current therapies, especially given the safety profile of anti-IL23 therapies. However, their results in treatment-naïve populations may not fully reflect the complexity of real-world patients who are treatment-experienced, many of whom require alternative strategies due to prior therapeutic failures. Future research, including studies on patients with bio failure, will be needed to bridge this gap.

## 9. Conclusion

Gastroenterologists are fortunate to have a wide variety of anti-IL23p19 therapies available for use in IBD, as well as in concomitant immune-mediated diseases like psoriasis and psoriatic arthritis. However, this poses a challenge in deciding on the most appropriate therapeutic administration of a specific anti-IL23p19 agent. In this review, we have tried to address this. Based on current evidence, several key considerations can be derived for the use of anti-IL23p19 agents in clinical practice.

The maintenance regimen of a single SC injection every 2 months following IV induction makes all 3 agents appealing to patients who prioritize convenience and autonomy in managing their treatment through self-injections. With SC GUS induction now approved for CD, and potentially for UC in the near future, eligible patients may benefit from the flexibility of choosing between IV and SC induction routes. However, SC therapy may not be suitable for all patients. Those with poor adherence or aversion to self-injections may be better managed with regular IV-based therapies.

Patient preference on the type of SC delivery device should also be considered. As each anti-IL23p19 agent has a distinct delivery system, patients should be counselled on available options to support personalized decision-making before the final choice and prior to starting the therapy.

Attention should also be paid to dosing anti-IL23p19 therapies across different clinical scenarios. For induction, both MIR and RIS dosing regimens differ between CD and UC, while GUS follows a uniform approach for both diseases. During maintenance, MIR dosing remains disease specific, whereas both GUS and RIS require dose adjustments based on induction response (in CD and UC for GUS, and UC for RIS). In patients with indeterminate colitis, the higher anti-IL23p19 may be preferable. Notably, GUS and RIS are currently approved for plaque psoriasis and psoriatic arthritis, in addition to their indications for IBD, highlighting the potential value of higher dosing in patients with these comorbidities to ensure adequate disease control.

Regarding anti-IL12/23-refractory patients, the results of the GUS and RIS trials suggest switching to these agents may be beneficial. Conversely, switching from an anti-IL23p19 agent to an anti-IL12/23 agent is not currently recommended. In conclusion, anti-IL23p19 therapies are emerging as highly promising treatment options for moderate-to-severe IBD.

## Data Availability

No new data were generated or analyzed in support of this research.

## Fundings

This research received no specific grant.
